# Exploring Causal Associations Between Plasma Metabolites and Autism Spectrum Disorder

**DOI:** 10.31083/AP48246

**Published:** 2025-11-14

**Authors:** Shangyun Shi, Ancha Baranova, Hongbao Cao, Fuquan Zhang

**Affiliations:** ^1^Department of Psychiatry, The Affiliated Brain Hospital of Nanjing Medical University, 210029 Nanjing, Jiangsu, China; ^2^School of Systems Biology, George Mason University, Fairfax, VA 22030, USA; ^3^Research Centre for Medical Genetics, 115478 Moscow, Russia; ^4^Institute of Neuropsychiatry, The Affiliated Brain Hospital of Nanjing Medical University, 210029 Nanjing, Jiangsu, China

**Keywords:** autism spectrum disorder, plasma metabolite, Mendelian randomization, causal association

## Abstract

**Background:**

In autism spectrum disorder (ASD), the human plasma metabolome is altered but the causal relationship between the levels of metabolites and ASD is unclear. We aimed to assess bidirectional causal associations between plasma metabolites and ASD.

**Methods:**

We investigated potential causal associations between the genetic variation contributing to the levels of metabolites and ASD via Mendelian randomization (MR) analyses. Genome-wide association study (GWAS) summary datasets were used in the study, including ASD (n = 46,350) and 871 plasma metabolite (n = 8299) datasets. We used druggability analysis to prioritize metabolites with therapeutic potential.

**Results:**

Our MR analysis identified 32 plasma metabolites whose levels were protective against the risk of ASD, including 5 alpha-androstan-3 alpha, 17 beta-diol disulfate (odds ratio (OR): 0.94, 95% CI: 0.90–0.97) and 11beta-hydroxyetiocholanolone glucuronide (OR: 0.95, 95% CI: 0.92–0.98). Additionally, 12 metabolites were found to be positively associated with the risk of ASD, including indoleacetylglutamine (OR: 1.04, 95% CI: 1.01–1.08) and sphingomyelin (d18:1/24:1, d18:2/24:0) (OR: 1.06, 95% CI: 1.01–1.11). Some metabolites may be regulated through drug intervention, including sphingomyelin, chiro-inositol, carotene diol (1)/(2), and glycerol. Genetic variation contributing to ASD may increase the abundance of five metabolites, including deoxycholic acid glucuronide (OR: 1.18, 95% CI: 1.03–1.34); meanwhile, the abundance of 27 metabolites, including stearoyl choline (OR: 0.80, 95% CI: 0.69–0.92) may be causally reduced.

**Conclusions:**

Our MR analysis uncovered bidirectional causal associations between certain plasma metabolites and ASD, suggesting that these metabolites could be biomarkers for ASD and paving the way for novel therapeutic targets in ASD phenotypes.

## Main Points

1. In autism spectrum disorder (ASD), alterations occur in the human plasma metabolome. However, the causal relationship between metabolite levels and ASD remains ambiguous.

2. Mendelian randomization (MR) studies that utilize genetic variants as instrumental variables are now widely used to infer causality from a genetic perspective.

3. Our research indicates that specific plasma metabolites play a role in increasing the risk of ASD. Conversely, ASD has the potential to influence the composition of metabolites.

## 1. Introduction

Autism spectrum disorder (ASD) is a lifelong neurodevelopmental disorder arising from a constellation of genetic and environmental factors, with a heritability rate of approximately 85%. It is characterized by severe impairment of social interaction and communication skills, accompanied by restricted, repetitive, and stereotyped behavior patterns and interests [1, 2]. The prevalence rate of ASD is approximately 1.5% and it is increasing annually, imposing a heavy economic burden on families and society [3].

Patients with ASD often present with various other conditions such as anxiety, attention-deficit/hyperactivity disorder, and depression, which increases the complexity of ASD diagnosis and treatment. Due to the lack of specific biomarkers or imaging tests, diagnosis of ASD relies on clinical assessment, behavioral observations, and standardized questionnaires. Clinical manifestations of ASD exhibit significant heterogeneity, thus posing additional problems [4].

ASD patients exhibit overall decreased cellular energy balance and insufficient mitochondrial energy reserves, which may contribute to cognitive impairments, language deficits, and energy metabolism abnormalities. ASD-associated mitochondrial dysfunction may lead to reduced synaptic neurotransmitter release, which is observed in GABAergic interneurons. Increased susceptibility to oxidative stress in ASD patients may be linked to altered antioxidant enzyme activity, resulting in mitochondrial dysfunction and impaired energy metabolism [5, 6]. Mitochondrial dysfunction may not only be comorbid with ASD but also potentially a contributing factor [7].

Metabolites are small molecules formed during biochemical reactions. These molecules may be either intermediates or end products such as amino acids, nucleotides, lipids, hormones, and exogenous substances participating in human metabolism. Levels of individual metabolites are influenced by inherited genetic variants, diet, and other environmental exposures [8]. Novel metabolomics platforms continue to emerge and their application is helping to both uncover the pathogenesis of human diseases and identify novel biomarkers for diagnoses and prognoses [9, 10].

Alterations in plasma or serum metabolite levels have been found in previous studies of neuropsychiatric conditions [11, 12]. Of note, Zhang *et al*. [13] identified an association between the levels of 38 plasma metabolites and an increased risk of dementia. A large-scale study involving 5283 patients with major depressive disorder (MDD) and 10,145 controls showed that the levels of 21 metabolites were significantly associated with depression [14]. Similarly, when the blood and urine samples of ASD patients were compared with those collected from normally developing children, significant differences in the abundance of various metabolites were uncovered. The potential for distinguishing individuals with ASD from normal children was independently identified for compounds belonging to a variety of metabolite classes, including amino acids, lipids, and xenobiotics [7, 15, 16].

For many metabolites, their serum levels are highly heritable [17, 18]. Understanding if their involvement in ASD is causal may be beneficial for understanding the pathogenesis of this disease and the development of potential strategies for its treatment.

To evaluate the causal effects of each risk factor on a certain outcome, Mendelian randomization (MR) employs genetic variants as instrumental variables. Like randomized assignment of individuals to the treatments in clinical trials, the random assortment of genetic variants in an individual’s diploid genome provides a framework to evaluate the causality of the observed associations between exposures and outcomes. These associations are inferred from aggregated data collected in genome-wide association studies (GWAS). This approach minimizes the effects of confounding factors, including age, other drug or environmental exposures, and reverse causation [19, 20], and now is widely used to infer causality from a genetic perspective [21, 22, 23, 24, 25]. In this study, we used MR analysis to explore the causal effects connecting various small molecular constituents of plasma and ASD. We then evaluated whether the identified ASD-related metabolites can be modulated by pharmacological or other interventions.

## 2. Methods

### 2.1 Data Sources and Study Design

The GWAS summary results used for this analysis were all derived from publicly available data. The GWAS data for ASD (n = 46,350; 18,381 cases and 27,969 controls) were sourced from the Integrative Psychiatric Research (iPSYCH) and Psychiatric Genomics Consortium (PGC). The PGC samples were retrieved from five European cohorts. The iPSYCH samples were gathered from a population-based cohort consisting of all children born in Denmark between 1981 and 2005 [26].

For metabolites, we focused on data from the Canadian Longitudinal Study of Aging (CLSA). The research targeted 8299 unrelated European subjects within the CLSA, all of whom underwent genome-wide genotyping and blood metabolite measurement. The CLSA tracks over 50,000 Canadians aged 45–85 years (50.9% female) for various types of information such as biological and medical data. The levels of blood metabolites were measured using the ultrahigh-performance liquid chromatography-tandem mass spectrometry (UPLC-MS/MS) platform by Metabolon Inc. (Morrisville, CA, USA) [27].

Among the plasma metabolites tested, those with known identities spanned eight super-pathways (lipids, amino acids, xenobiotics, nucleotides, cofactors and vitamins, carbohydrates, peptides, and energy). After excluding the metabolites labeled as “X-” (unknown), we tested 871 metabolites. When analyzing the metabolic pathways, we referred to the original research literature, which contained the metabolic pathways of these metabolites. Ethical approval was obtained in all original studies. A flowchart outlining the current study is shown in **Supplementary Fig. 1**.

### 2.2 Instrumental Variables (IVs)

We selected candidate instrumental variables (IVs) based on a genome-wide significance threshold of *p* < 1.0 × 10^-5^. Single nucleotide polymorphisms (SNPs) were selected using the 1000 Genomes Project Phase 3 (EUR) reference panel [28] and, to ensure the independence of the IVs, we performed pruning with an r^2^ threshold of 0.001 within a 10 Mb window. This strategy helped to ensure that the IVs included in the analysis were independent, thereby improving the precision and robustness of the MR results.

### 2.3 MR Analysis

In this study, we utilized three different models from the TwoSampleMR package (https://mrcieu.github.io/TwoSampleMR) in the R software (R Core Team, Vienna, Austria, version 4.0.5) to investigate causal relationships [29]. We primarily utilized the inverse-variance-weighting (IVW) model, which assumes a zero intercept and estimates causal effects through fixed-effect meta-analysis. To ensure the robustness of our findings, we conducted sensitivity analyses using additional MR methods, such as the weighted median (WM) and MR-Egger methods, which differ from IVW in their assumptions. The latter model, for instance, assumes that pleiotropic effects are independent and employs weighted linear regression of outcome coefficients against exposure coefficients. We assessed the presence of pleiotropy by examining the intercepts of MR-Egger regression, which provided insights into mean-level pleiotropy. Furthermore, we evaluated the heterogeneity of our results using both I^2^ statistics and Cochran’s Q test, considering values where both I^2^ > 0.25 and P_Q_ < 0.05 are indicative of significant heterogeneity. An IVW-based *p* < 0.05 indicated a significant correlation between exposure and outcome.

### 2.4 Druggability Evaluation

We searched for targets and drug information in DrugBank [30] and ChEMBL [31] to assess whether the identified metabolites could serve as potential therapeutic targets. These databases prioritized druggable targets by integrating information from text mining, gene function, drug-gene interactions, and expert curation.

## 3. Results

### 3.1 Selection of Instrumental Variables

Following an extensive quality control procedure, we discovered a total of 18,669 SNPs associated with metabolites as IVs for investigating the association between metabolites and ASD. In the reverse MR analysis, we included a total of 54 IVs associated with ASD. Detailed descriptors of these SNPs are shown in **Supplementary Tables 1,2**.

### 3.2 MR Analysis

**Fig. 1.**
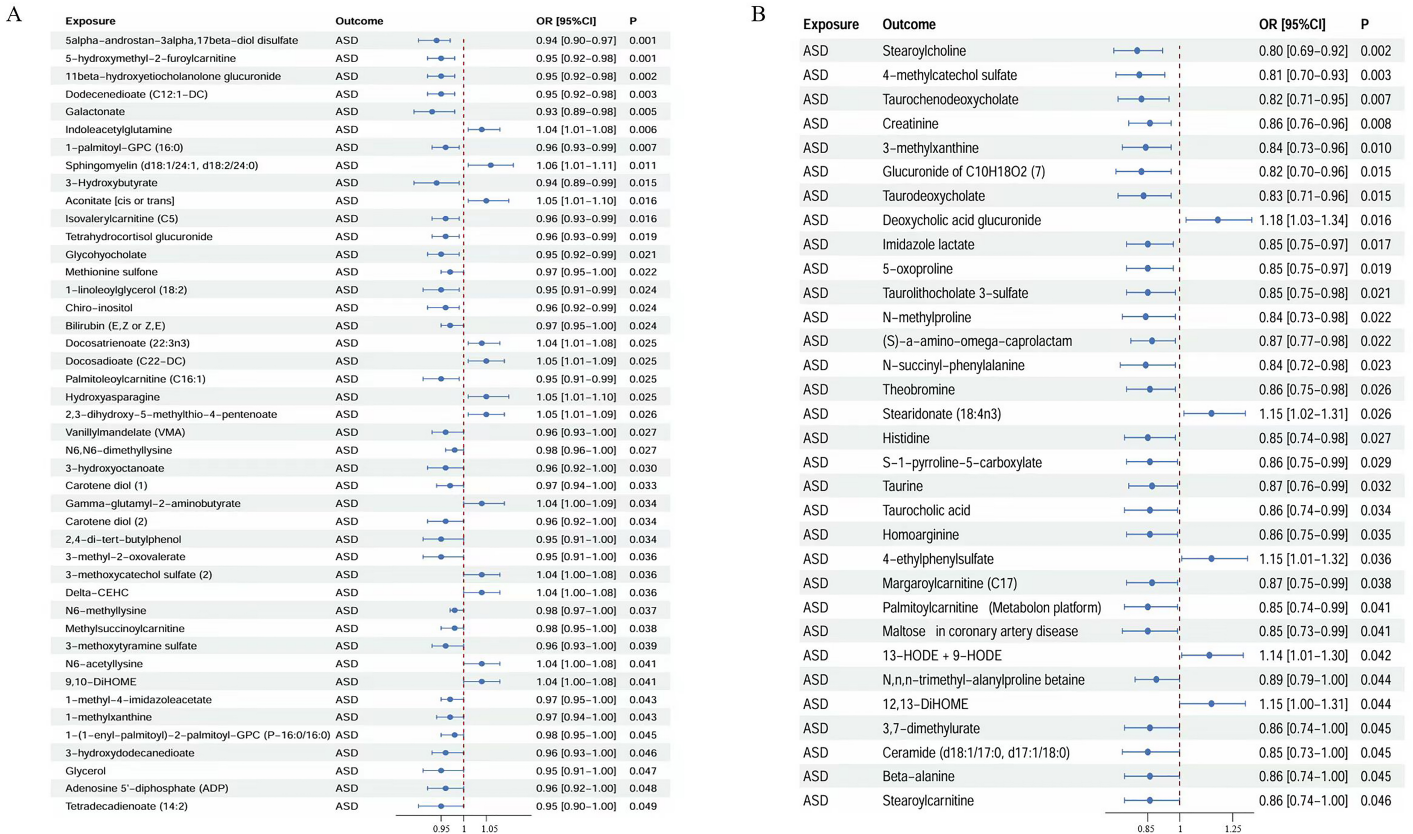
**Bidirectional causal effects between plasma metabolites and ASD**. (A) Causal effects of plasma metabolites on ASD. (B) Causal effects of ASD on plasma metabolites. CI, confidence interval; ASD, autism spectrum disorder; OR, odds ratio.

**Fig. 2.**
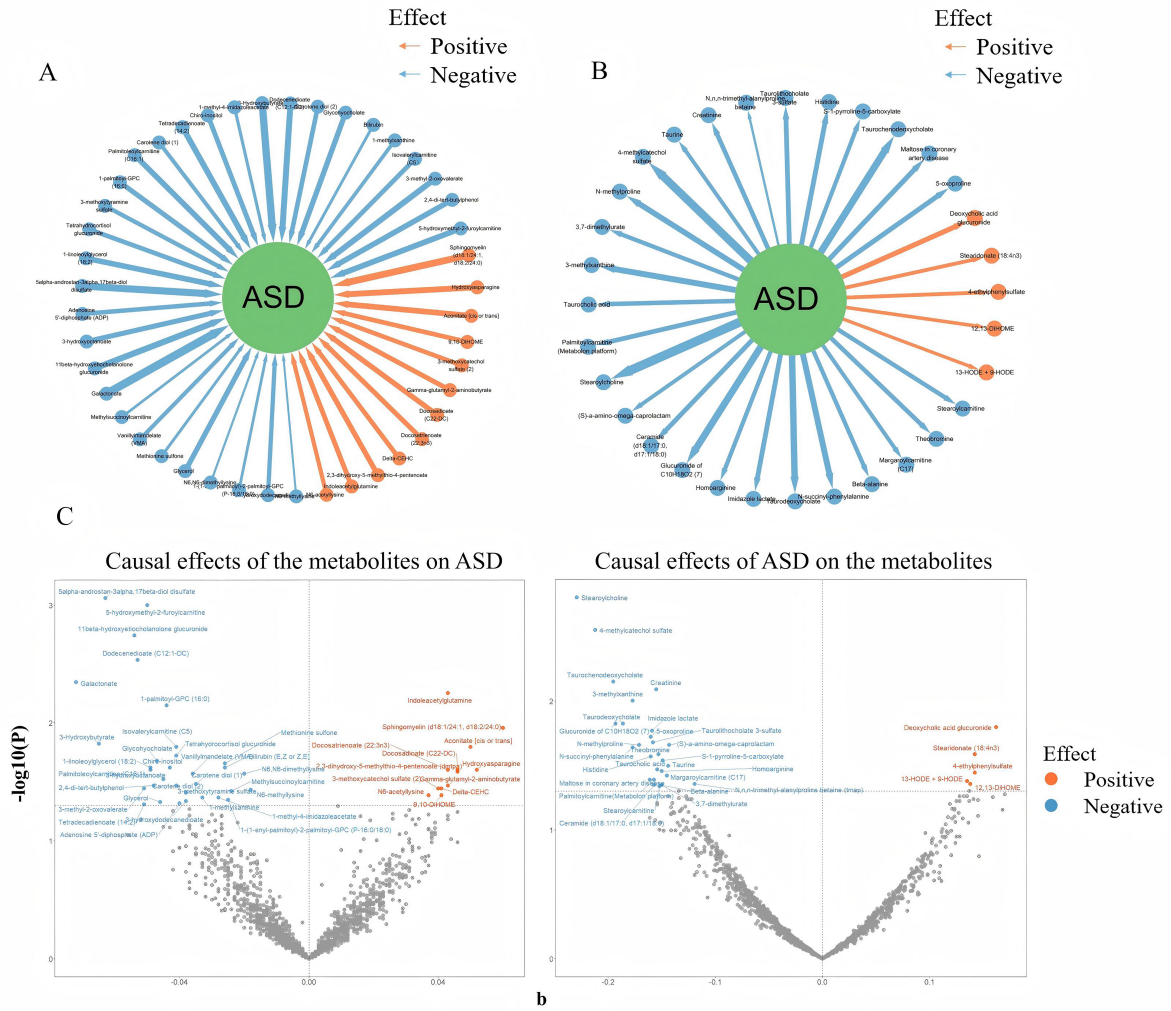
**Causal effects between plasma metabolites and ASD**. (A,B) Overview of plasma metabolite and ASD regulatory network. The arrow points from exposure to outcome; line thickness indicates the effect size. (C) Volcano plot of causal effects between plasma metabolites and ASD. Line colors indicate different effects: red, positive effect; blue, negative effect. b, refers to beta, which represents the effect size.

Using IVW, a total of 44 metabolites were identified as conferring causal effects on ASD (Table 1 and Fig. 1A). Of these, 12 were found to be significantly and positively associated with the risk of ASD, including indoleacetylglutamine and sphingomyelin (d18:1/24:1, d18:2/24:0). Conversely, 32 metabolites, including 5alpha-androstan-3alpha,17beta-diol disulfate and 11beta-hydroxyetiocholanolone glucuronide, were highlighted as protective factors against ASD (positive effects shown in red and negative effects shown in blue in Fig. 2A–C).

**Table 1.** **MR analyses reveal causal effects of plasma metabolites on ASD**.

Exposure	Outcome	OR [95% CI]	*p*
5alpha-androstan-3alpha,17beta-diol disulfate	ASD	0.94 [0.90–0.97]	8.72 × 10^-4^
5-hydroxymethyl-2-furoylcarnitine	ASD	0.95 [0.92–0.98]	1.00 × 10^-3^
11beta-hydroxyetiocholanolone glucuronide	ASD	0.95 [0.92–0.98]	1.81 × 10^-3^
Dodecenedioate (C12:1-DC)	ASD	0.95 [0.92–0.98]	2.92 × 10^-3^
Galactonate	ASD	0.93 [0.89–0.98]	4.51 × 10^-3^
Indoleacetylglutamine	ASD	1.04 [1.01–1.08]	5.58 × 10^-3^
1-palmitoyl-GPC (16:0)	ASD	0.96 [0.93–0.99]	7.08 × 10^-3^
Sphingomyelin (d18:1/24:1, d18:2/24:0)	ASD	1.06 [1.01–1.11]	0.011
3-Hydroxybutyrate	ASD	0.94 [0.89–0.99]	0.015
Aconitate [cis or trans]	ASD	1.05 [1.01–1.10]	0.016
Isovalerylcarnitine (C5)	ASD	0.96 [0.93–0.99]	0.016
Tetrahydrocortisol glucuronide	ASD	0.96 [0.93–0.99]	0.019
Glycohyocholate	ASD	0.95 [0.92–0.99]	0.021
Methionine sulfone	ASD	0.97 [0.95–1.00]	0.022
1-linoleoylglycerol (18:2)	ASD	0.95 [0.91–0.99]	0.024
Chiro-inositol	ASD	0.96 [0.92–0.99]	0.024
Bilirubin (E, Z or Z, E)	ASD	0.97 [0.95–1.00]	0.024
Docosatrienoate (22:3n3)	ASD	1.04 [1.01–1.08]	0.025
Docosadioate (C22-DC)	ASD	1.05 [1.01–1.09]	0.025
Palmitoleoylcarnitine (C16:1)	ASD	0.95 [0.91–0.99]	0.025
Hydroxyasparagine	ASD	1.05 [1.01–1.10]	0.025
2,3-dihydroxy-5-methylthio-4-pentenoate	ASD	1.05 [1.01–1.09]	0.026
Vanillylmandelate (VMA)	ASD	0.96 [0.93–1.00]	0.027
N6, N6-dimethyllysine	ASD	0.98 [0.96–1.00]	0.027
3-hydroxyoctanoate	ASD	0.96 [0.92–1.00]	0.030
Carotene diol (1)	ASD	0.97 [0.94–1.00]	0.033
Gamma-glutamyl-2-aminobutyrate	ASD	1.04 [1.00–1.09]	0.034
Carotene diol (2)	ASD	0.96 [0.92–1.00]	0.034
2,4-di-tert-butylphenol	ASD	0.95 [0.91–1.00]	0.034
3-methyl-2-oxovalerate	ASD	0.95 [0.91–1.00]	0.036
3-methoxycatechol sulfate (2)	ASD	1.04 [1.00–1.08]	0.036
Delta-CEHC	ASD	1.04 [1.00–1.08]	0.036
N6-methyllysine	ASD	0.98 [0.97–1.00]	0.037
Methylsuccinoylcarnitine	ASD	0.98 [0.95–1.00]	0.038
3-methoxytyramine sulfate	ASD	0.96 [0.93–1.00]	0.039
N6-acetyllysine	ASD	1.04 [1.00–1.08]	0.041
9,10-DiHOME	ASD	1.04 [1.00–1.08]	0.041
1-methyl-4-imidazoleacetate	ASD	0.97 [0.95–1.00]	0.043
1-methylxanthine	ASD	0.97 [0.94–1.00]	0.043
1-(1-enyl-palmitoyl)-2-palmitoyl-GPC (P-16:0/16:0)	ASD	0.98 [0.95–1.00]	0.045
3-hydroxydodecanedioate	ASD	0.96 [0.93–1.00]	0.046
Glycerol	ASD	0.95 [0.91–1.00]	0.047
Adenosine 5^′^-diphosphate (ADP)	ASD	0.96 [0.92–1.00]	0.048
Tetradecadienoate (14:2)	ASD	0.95 [0.90–1.00]	0.049

GPC, glycerophosphocholine; CEHC, carboxyethylhydroxychroman.

Reverse MR analysis revealed that genetic liability to ASD is linked to increased levels of five metabolites, including deoxycholic acid glucuronide and stearidonate (18:4n3), as well as a reduction in 27 metabolites such as stearoyl choline, 4-methylcatechol sulfate, and taurine (Table 2; Fig. 1B; positive effects shown in red and negative effects shown in blue in Fig. 2B,C).

**Table 2.** **MR analyses reveal causal effects of ASD on plasma metabolites**.

Exposure	Outcome	OR [95% CI]	*p*
ASD	Stearoylcholine	0.80 [0.69–0.92]	1.57 × 10^-3^
ASD	4-methylcatechol sulfate	0.81 [0.70–0.93]	2.81 × 10^-3^
ASD	Taurochenodeoxycholate	0.82 [0.71–0.95]	7.08 × 10^-3^
ASD	Creatinine	0.86 [0.76–0.96]	8.12 × 10^-3^
ASD	3-methylxanthine	0.84 [0.73–0.96]	9.94 × 10^-3^
ASD	Glucuronide of C_10_H_18_O_2_ (7)	0.82 [0.70–0.96]	0.015
ASD	Taurodeoxycholate	0.83 [0.71–0.96]	0.015
ASD	Deoxycholic acid glucuronide	1.18 [1.03–1.34]	0.016
ASD	Imidazole lactate	0.85 [0.75–0.97]	0.017
ASD	5-oxoproline	0.85 [0.75–0.97]	0.019
ASD	Taurolithocholate 3-sulfate	0.85 [0.75–0.98]	0.021
ASD	N-methylproline	0.84 [0.73–0.98]	0.022
ASD	(S)-a-amino-omega-caprolactam	0.87 [0.77–0.98]	0.022
ASD	N-succinyl-phenylalanine	0.84 [0.72–0.98]	0.023
ASD	Theobromine	0.86 [0.75–0.98]	0.026
ASD	Stearidonate (18:4n3)	1.15 [1.02–1.31]	0.026
ASD	Histidine	0.85 [0.74–0.98]	0.027
ASD	S-1-pyrroline-5-carboxylate	0.86 [0.75–0.99]	0.029
ASD	Taurine	0.87 [0.76–0.99]	0.032
ASD	Taurocholic acid	0.86 [0.74–0.99]	0.034
ASD	Homoarginine	0.86 [0.75–0.99]	0.035
ASD	4-ethylphenylsulfate	1.15 [1.01–1.32]	0.036
ASD	Margaroylcarnitine (C17)	0.87 [0.75–0.99]	0.038
ASD	Palmitoylcarnitine (Metabolon platform)	0.85 [0.74–0.99]	0.041
ASD	Maltose in coronary artery disease	0.85 [0.73–0.99]	0.041
ASD	13-HODE + 9-HODE	1.14 [1.01–1.30]	0.042
ASD	N,n,n-trimethyl-alanylproline betaine	0.89 [0.79–1.00]	0.044
ASD	12,13-DiHOME	1.15 [1.00–1.31]	0.044
ASD	3,7-dimethylurate	0.86 [0.74–1.00]	0.045
ASD	Ceramide (d18:1/17:0, d17:1/18:0)	0.85 [0.73–1.00]	0.045
ASD	Beta-alanine	0.86 [0.74–1.00]	0.045
ASD	Stearoylcarnitine	0.86 [0.74–1.00]	0.046

HODE, hydroxyoctadecadienoic acid.

The results showed that metabolites were mostly concentrated at the lipid and amino acid levels, and the metabolic pathways involved included androgenic steroid biosynthesis, tryptophan metabolism, primary bile acid metabolism, creatine metabolism, and purine metabolism. For details on other relevant metabolic pathways, see **Supplementary Table 3**.

Except for adenosine 5^′^-diphosphate (P_Q_ < 0.05), Cochrane’s Q-test indicated that there was no significant heterogeneity between most SNPs. However, the MR-Egger model analysis detected some horizontal pleiotropy involving adenosine 5^′^- diphosphate, glucuronide of C_10_H_18_O_2_ (7), and deoxycholic acid glucuronide (*p*_pleiotropy < 0.05), amongst others. Therefore, these results should be interpreted with caution (**Supplementary Tables 4,5**).

### 3.3 Five Metabolites as Therapeutic Drug Targets

Druggability evaluation showed that five of the ASD-related metabolites (sphingomyelin, chiro-inositol, carotene diol (1)/(2), and glycerol) have been targeted by pharmacologic intervention. One of these drugs, olipudase alfa, that targets sphingomyelin (d18:1/24:1, d18:2/24:0) has been used to treat acid sphingomyelinase deficiency by catalyzing the hydrolysis of sphingomyelin, thereby reducing its accumulation. Inositol and beta-carotene are used as nutritional supplements in special dietary foods and infant formula. Inositol plays an important role in ensuring oocyte fertility, and its association with D-chiro inositol has also been studied in polycystic ovary syndrome. The use of glycerin may help to improve gastrointestinal symptoms in ASD patients (**Supplementary Table 6**).

## 4. Discussion

In this study, we explored potential associations between human plasma metabolites and ASD by conducting a two-sample MR analysis on GWAS summary statistics datasets. We identified 44 metabolites with underlying genetic variation causally related to ASD risk. Druggability evaluation prioritized five ASD-related metabolites (sphingomyelin, chiro-inositol, carotene diol (1)/(2), and glycerol) that could be modified by drug interventions. In addition, the genetic component of ASD affected the levels of 32 metabolites.

Previous research has shown that there are significant differences in the metabolic profiles between children with ASD and neurotypical children. These differences extensively involve various metabolic pathways that involve lipids, amino acids, nucleotides, and energy. These differences may change with age [15, 32, 33]. Liu *et al*. [34] conducted a quantitative analysis of 28 children with ASD and identified three potential biomarkers. The findings of our study demonstrate that the metabolites are primarily related to lipids and amino acids. The metabolic pathways involved include androgenic steroid biosynthesis, tryptophan metabolism, primary bile acid metabolism, creatine metabolism, purine metabolism, methionine, cysteine, S-Adenosylmethionine (SAM), and taurine metabolism.

An imbalance in oxidative stress responses and pro-inflammatory processes [35] is a commonly observed feature in patients with ASD, who often exhibit metabolic abnormalities associated with mitochondrial dysfunction, affecting cellular energy production [36, 37, 38, 39]. The tricarboxylic acid (TCA) cycle occurs in the mitochondrial matrix and is crucial for many cellular processes. Our research indicates that the genetic signature contributing to the elevation of the levels of cis-aconitate, a TCA intermediate, raises the risk of ASD. Sotelo-Orozco *et al*. [40] demonstrated that an increase in cis-aconitate levels is linked to poorer cognitive and adaptive skills in children with ASD. Likhitweerawong *et al*. [16] also confirmed that cis-aconitate was significantly elevated in patients with ASD.

Moreover, the activity of aconitase, an enzyme that degrades cis-aconitate, is lower in the autism cerebellum and is negatively correlated with the reduced glutathione/oxidized glutathione (GSH/GSSG) ratio, which indicates glutathione redox/antioxidant capacity [41]. GSH plays a protective role against oxidative stress and neuroinflammation. Depletion of GSH following chronic gastrointestinal issues, which are common in ASD patients, is also associated with mitochondrial dysfunction [42, 43]. Thus, it is not surprising that our reverse MR analysis showed that the lowering of the levels of 5-oxoproline, a major intermediate in the γ-glutamyl cycle whereby glutathione is produced and then broken down, is also associated with ASD occurrence.

A study involving 20 Egyptian children aged 2–7 years found that the severity of autism symptoms, measured by the Childhood Autism Rating Scale (CARS), negatively correlates with levels of leucine, isoleucine, phenylalanine, cysteine, serine, and tyrosine [40]. Notably, the absence of the solute carrier transporter 7a5 (SLC7A5) at the blood-brain barrier leads to a significant reduction in the levels of large neutral amino acids (LNAAs) in the brain parenchyma, possibly contributing to the pathogenesis of ASD [44]. Moreover, the metabolism of LNAAs and lipids are interconnected, with the deletion of SLC7A5 in the neurons affecting their metabolic state, leading to a shift in lipid metabolism [45].

Glutamatergic dysfunction has been implicated in the pathophysiology of ASD, with multiple studies reporting abnormalities in glutamate levels across different regions of the ASD brain [46, 47]. Our reverse MR analysis indicated that a reduction in S-1-pyrroline-5-carboxylate, involved in glutamate metabolism, is associated with the risk of ASD. On the other hand, the genetic signature predisposing to ASD is associated with a decrease in the levels of taurine, a gliotransmitter and an antioxidant. A previous study connected deficiencies in hypotaurine and taurine production with defects in cell differentiation in the brain [48]. ASD-related taurine pathway abnormalities may further impact neuronal signaling and perpetuate a vicious metabolic circle supporting the pathophysiology of ASD [49, 50]. Notably, Likhitweerawong *et al*. [16] reported that taurine and homoarginine were elevated in ASD patients, which is contrary to our results.

In addition to the alterations in amino acid levels, which are evident in individuals with ASD and require further research, a variety of lipid abnormalities were detected [51]. In particular, our study highlights the possible causality of ASD-associated changes in fatty acid metabolism. Long-chain polyunsaturated fatty acids (PUFAs) such as arachidonic and docosahexaenoic acids are known to influence neurological responses and behavior [16, 52, 53, 54]. Our research indicates that docosatrienoate (22:3n3), a biomarker of exposure to the polychlorinated biphenyl (PCB) pollutants [55], and the relatively uncharacterized tetradecadienoate (14:2), both belonging to the category of long-chain PUFAs, exert opposite causal effects on the risks of ASD. Additionally, these risks were associated with genetic signature contributing to increased levels of stearidonate (18:4n3), which is a derivative of α-linolenic acid and also elevated after exposure to PCB and in patients with hypertension [56]. Levels of various PUFAs fluctuate across different cohorts or populations, possibly due to differences in lifestyle habits, geographic location, and other factors [37, 57]. For some long-chain saturated fatty acids, such as margaroylcarnitine, Needham *et al*. [7] also pointed out that there were significant differences between ASD patients and the control group. Moreover, 4-ethylphenyl sulfate was significantly increased in ASD mice. In the plasma of ASD patients, the levels of saturated fatty acid and long-chain acylcarnitines are generally lower than those in matched controls, which is consistent with our findings [7]. These findings suggest that dysregulation in fatty acid metabolism may play a role in the development of ASD [58, 59].

Another important class of metabolites with levels elevated in patients with ASD is derived from steroids, such as androgens and pregnenolone, and these levels correlate with the severity of ASD [59, 60]. In our study, genetic signatures promoting an increase in the levels of two androgenic steroids, 5alpha-androstan-3alpha,17beta-diol disulfate and 11beta-hydroxyetiocholanolone glucuronide, were protective against the risk of ASD. In trials of antioxidant therapy for ASD children, an increase in the levels of certain androgens has been associated with behavioral improvements [61]. Future research needs to further determine the relationship between changes in the levels of individual species of polyunsaturated and steroid lipids and ASD.

The identified metabolites can be regulated through pharmacological interventions or modifiable factors. Specifically, drugs used to treat conditions such as acid sphingomyelinase deficiency or polycystic ovary syndrome can regulate metabolites (sphingomyelin and inositol). In addition, the use of carotene diol or glycerol may be effective in improving symptoms in ASD patients.

Our study has several limitations. MR analysis may introduce biases due to various effects, so we employed multiple models to test the hypotheses. We did not correct for multiple testing on each *p*-value, which may have increased the risk of false positives. Additionally, some results suggest the presence of potential horizontal pleiotropy, which requires further validation. Our analysis relied solely on genetic factors, so caution is needed when interpreting the results. Furthermore, our data predominantly came from European populations, which may limit the generalizability of the findings. While our study identified certain metabolites associated with ASD risk, this primarily offers predictive insights that require validation. Future research should comprehensively explore the causal relationships and potential molecular mechanisms.

## 5. Conclusions

Our study suggests that certain plasma metabolites contribute to the risk of ASD, while ASD may affect the composition of the metabolites. These findings suggest the potential for developing new diagnostic tools and therapeutic targets to optimize ASD diagnosis and management.

## Availability of Data and Materials

All data generated or analyzed during this study are included in this published article and its supplementary information files.

## Supplementary Material

Supplementary material associated with this article can be found, in the online version, at https://doi.org/10.31083/AP48246.
