# Supplementary figures and images for: Exploring Causal Associations Between Plasma Metabolites and Autism Spectrum Disorder
Source: Alpha Psychiatry. 2025 Nov 14;26(6):48246. doi: 10.31083/AP48246 (PMC12781214; doi:10.31083/AP48246)

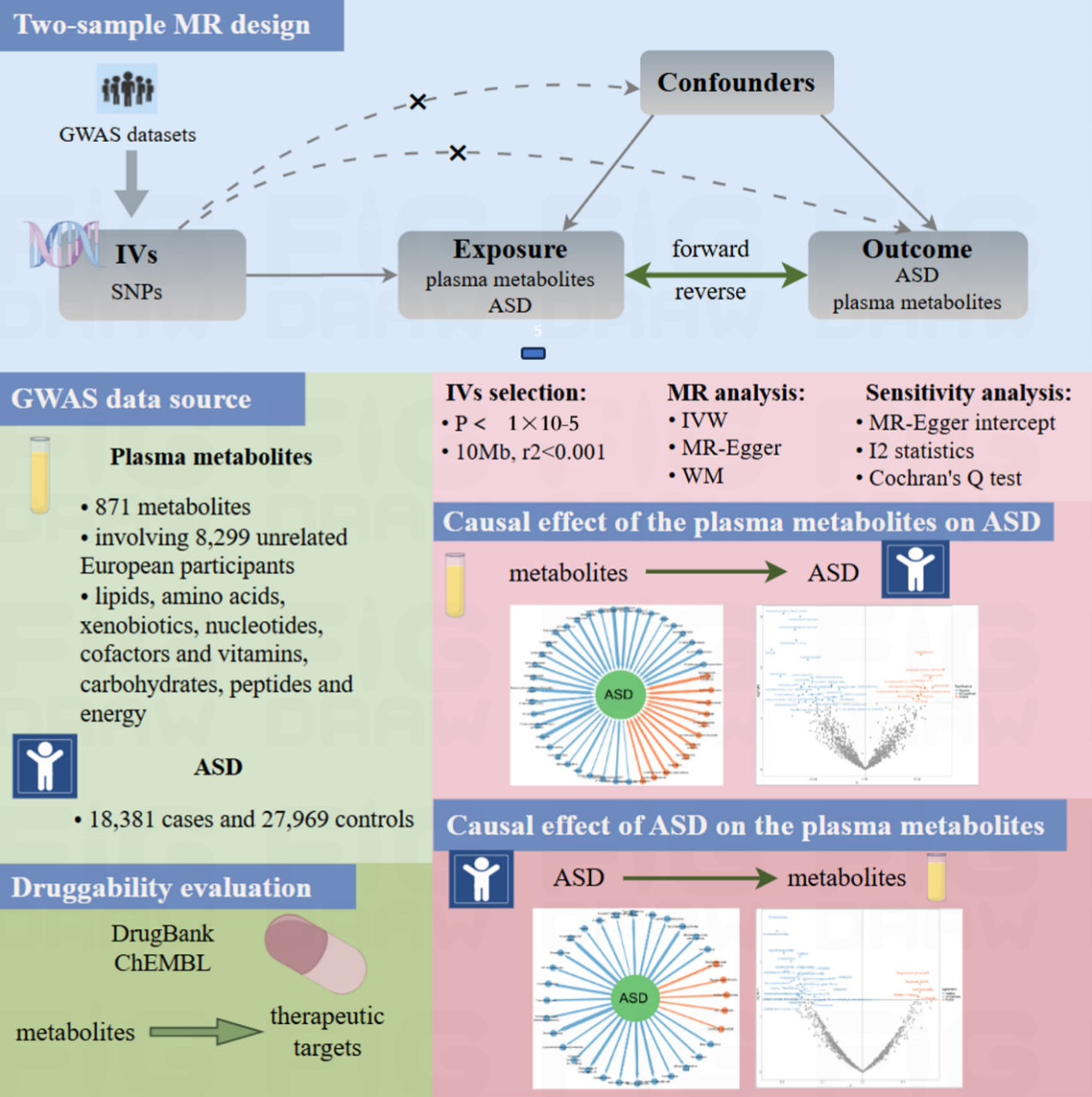


Supplementary Fig. 1

Supplement: Supplementary file 1 [file 2757-8038-26-6-48246-s1.zip › Supplementary Fig. 1.docx]

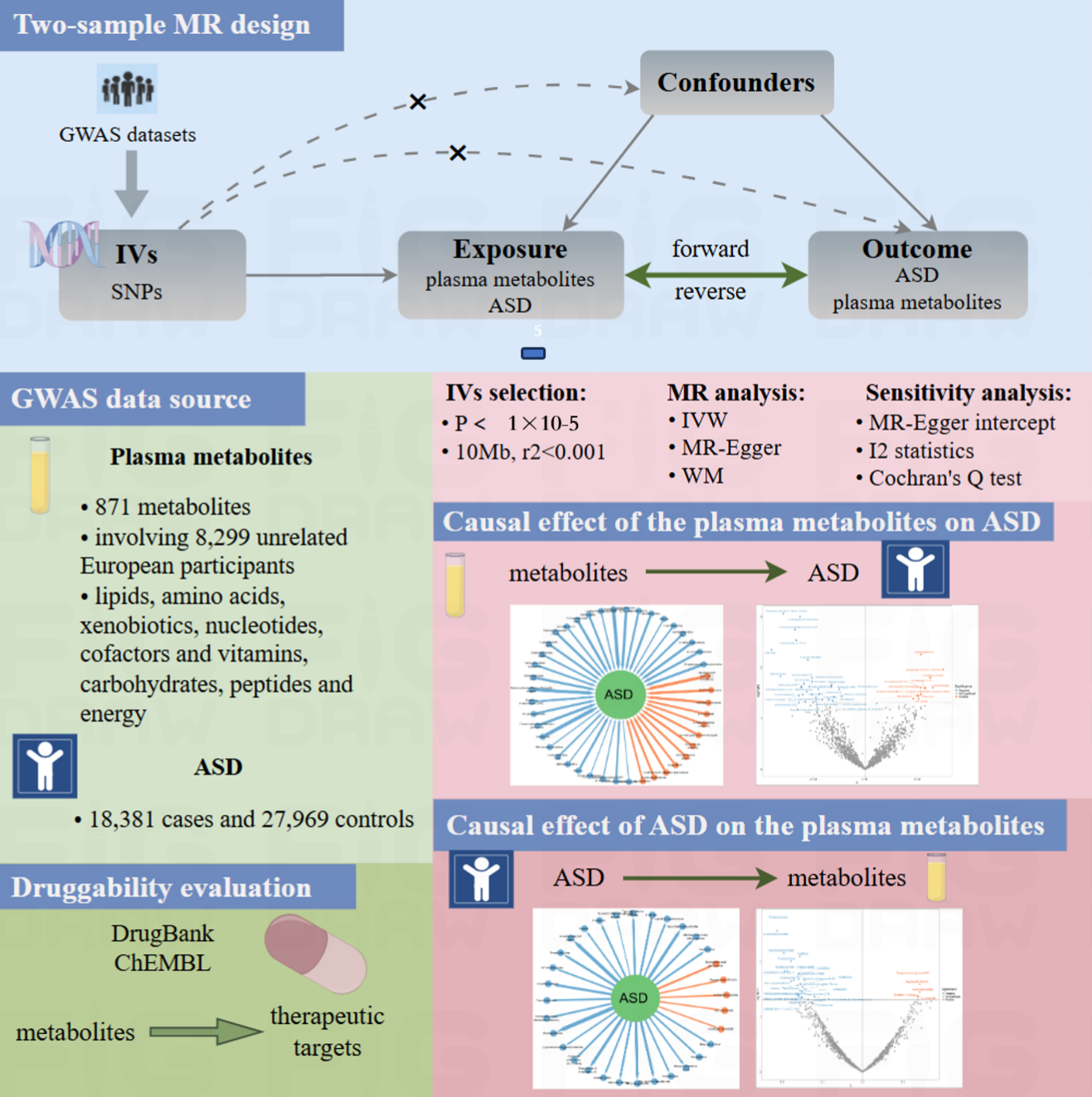

Supplement: Supplementary file 1 [file 2757-8038-26-6-48246-s1.zip › Supplementary Fig. 1.png]
